# Cadmium and Lead Content in Chosen Commercial Fishery Products Consumed in Poland and Risk Estimations on Fish Consumption

**DOI:** 10.1007/s12011-017-1104-1

**Published:** 2017-07-27

**Authors:** Anna Winiarska-Mieczan, Mariusz Florek, Małgorzata Kwiecień, Katarzyna Kwiatkowska, Robert Krusiński

**Affiliations:** 1 Department of Bromatology and Food Physiology, University of Life Sciences in Lublin, Akademicka 13, 20-950 Lublin, Poland; 2 Department of Commodity Science and Processing of Animal Raw Materials, University of Life Sciences in Lublin, Lublin, Poland

**Keywords:** Fish, Fishery products, Cadmium, Lead, Safety

## Abstract

The presented studies aimed to verify whether fish and fishery products available on the Polish market were safe for consumers in terms of Cd and Pb content. Safety was evaluated according to the content of Cd and Pb in fishery products and based on the share of such products in supplying Cd and Pb in the weekly diet of an adult. Fish samples, of which 139 were smoked fish (26 samples of mackerel, 21 of salmon, 35 of sprat, 38 of eel and 19 of trout) and 117 samples of prepared fish-based dishes (20 of salads, 41 of spreads and 56 of marinated herring) were analysed. The content of Cd and Pb was determined using the GF AAS method. The content of Cd per 1 kg of the analysed product can be represented as follows: salads > smoked eel > smoked salmon and mackerel > smoked trout and spreads > marinated herring > smoked sprat. The content of Pb per 1 kg of the analysed product can be represented as follows: smoked salmon and salads > smoked mackerel and spreads > smoked eel > smoked sprat and smoked trout. Most Cd was found in salads (on average 10.7 μg kg^−1^; range 6.53–14.7 μg), whereas most Pb was recorded in salads (on average 56.8 μg per kg; range 32.6–78.9 μg) and marinated fish (on average 58.8 μg per kg; range 19.8–79.6 μg). Fish and fishery products available in Poland are safe for consumers.

## Introduction

Cadmium (Cd) and lead (Pb) are toxic metals that are most common in the natural environment. They display mutagenic, teratogenic, genotoxic and carcinogenic effects [1]. The metals are capable of accumulating in tissues and are characterised by a long half-life: 5–30 years for Cd, and for Pb 30 days (in soft tissues) or even more than 10 years (in bones) [2, 3]. Cadmium has a particularly toxic effect on the liver and kidneys [4], whereas the toxicity of Pb mostly affects the cardiovascular system, excretory system (kidneys) and the central nervous system [5]. Chronic exposure to low doses of Cd and Pb is exceptionally hazardous to the body since these metals have no threshold level of toxicity.

Water reservoirs are most exposed to pollution since they receive pollutants from various sources: industrial effluent and municipal wastewater, agricultural contaminants (from runoffs), and airborne harmful substances (from rainfall) [6]. Fish constitute one of the last cells within the trophic network of aqueous ecosystems so significant amounts of toxic metals accumulate in their tissues [7]. The degree of Cd and Pb contamination in fish is determined by their habitat (depending on the degree of water pollution), duration of exposure to pollution and the eating habits of the fish [8]. It is a known fact that predators accumulate much more toxic metals in their tissues since the metals are capable of accumulating in the organism and are thus transmitted to a higher level of the food chain. Therefore, the likelihood that fish will be contaminated with heavy metals increases with their age. Metals are primarily accumulated in the kidneys, gills and liver of fish [9].

The content of toxic metals and other hazardous substances in fish is not a reason to make these products inadvisable for consumption, primarily due to the fact that for humans, fish is the only actual source of long-chain fatty acids from the n-3 group preventing cardiovascular diseases [10]. However, consumption of some species of fish (mainly predatory ones) should be limited in the first place by particularly sensitive people, e.g. young children or expectant and breastfeeding women [11]. According to data available in the Statistical Yearbook of the Republic of Poland [12] in 2015, the monthly intake of fish and seafood amounted to 0.33 kg per person. However, this figure was not inclusive of fishery products, including semi-prepared products and frozen and breaded fish the intake of which accounts for ca. 70% of the intake of all fishery products in Poland. The annual intake of fish and fishery products in Poland is about 5.8 kg per person (ca. 112 g per person per week) [13], whereas the recommended weekly intake is ca. 300 g per person [14]. The presented studies aimed to verify whether fish and fishery products available in the Polish market were safe for consumers in terms of Cd and Pb content. Safety was evaluated according to the content of Cd and Pb in fish and fishery products and based on the share of such products in supplying Cd and Pb in the weekly diet of an adult. The presented studies form part of a project aiming to estimate the exposure of the Polish population to the intake of Cd and Pb with food.

## Material and Methods

### Study Material

Fish samples, of which 139 were smoked fish (26 samples of mackerel, 21 of salmon, 35 of sprat, 38 of eel and 19 of trout) and 117 samples of prepared fish-based dishes (20 of salads, 41 of spreads and 56 of marinated fish) purchased from local groceries were analysed (Table 1). All the products were labelled with information that the fish were raised in fisheries meeting Marine Stewardship Council (MSC) standards for well-managed sustainable fishing. Meat was separated from the products containing inedible parts (skin, heads, fins, bones). The liquid in fish salads was not separated. The sample products were flaked using mechanical methods (MPW-02 apparatus equipped with plastic cutters, MPW, Poland), and test samples were taken (~20 g), placed in plastic containers, and frozen (−40 °C) for future chemical analysis.

**Table 1:** Characteristic of analysed fishery products

	Number	Percent of fish (%)	Fish species	Type of packaging
Smoked	139			
Mackerel A	11	100	Mackerel	Plastic containers
Mackerel B	15	100	Mackerel	Not packaged
Salmon A	9	100	Salmon	Plastic containers
Salmon B	12	100	Salmon	Not packaged
Sprat A	20	100	Sprat	Plastic containers
Sprat B	15	100	Sprat	Not packaged
Eel A	16	100	Eel	Plastic containers
Eel B	22	100	Eel	Not packaged
Trout A	10	100	Trout	Plastic containers
Trout B	9	100	Trout	Not packaged
Dishes	117			
Salad A	3	30–43	Mackerel	Plastic containers
Salad B	1	40	Sprat	Plastic containers
Salad C	6	22–55	Trout	Plastic containers
Salad D	8	30–50	Trout	Not packaged
Salad E	2	23–26	Tuna	Plastic containers
Spread A	3	23–24	Salmon	Plastic containers
Spread B	12	46–50	Salmon	Metal can
Spread C	16	23–24	Mackerel	Plastic containers
Spread D	10	30–35	Tuna	Plastic containers
Marinated A	15	40–90	Herrings	Plastic containers
Marinated B	25	40–85	Herring	Glass containers
Marinated C	16	60–90	Herring	Not packaged

### Analytical Procedure

The samples were thawed at room temperature and mixed manually. The procedure consisted of three steps: drying at 65 °C for 24 h and at 105 °C for a subsequent 24 h; ashing at 550 °C for 12 h with H_2_O_2_ used as an oxidant; dissolving in 10 ml of 1 M HNO_3,_ as described previously [15, 16]. The solutions were analysed by graphite furnace atomic absorption spectroscopy GF AAS in a Varian Spectr AA 880 equipped with a graphite furnace as presented in Table 2. The correctness of determination was verified by blank sample (1 M HNO_3_) and fish protein certified reference material for trace metals DORM-3 with the Cd content amounting to 0.290 mg per kg and Pb 0.395 mg per kg.

**Table 2:** Characteristic of parameters for the determination of Pb and Cd by GF AAS

	Cd	Pb
Wave length (nm)	228.8	217.0
Lamp current (mA)	4	10
Spectral band pass (nm)	0.5	1.0
LOD (mg kg^−1^)	0.001	0.011
LOQ (mg kg^−1^)	0.004	0.03
Pure gas	Argon	Argon
Background correction	Zeeman	Zeeman
The deviation of duplicate measurement (%)	5.8	4.5
Reproducibility (%)	10.1	13.9
Quality control		
Blank sample	1 M HNO_3_	1 M HNO_3_
Certified reference material	DORM-3 (fish protein)	DORM-3 (fish protein)
Certified element concentration in DORM-3		
Certified (mg kg^−1^)	0.290	0.395
Observed (mg kg^−1^)	0.281	0.374
Recovery rate (%)	97	95

### Chemical Reagents

HNO_3_ and H_2_O_2_ were purchased from POCH S.A. (Poland), while the standard solutions of Cd (1000 mg Cd as CdCl_2_ per 1 L H_2_0) and Pb (1000 mg Pb as Pb(NO_3_)_2_ per 1 L H_2_O) used to draw the calibration curve were purchased from Merck (Germany). Fish protein-certified reference material for trace metals DORM-3 was purchased from the National Research Council Canada.

### Calculation and Statistical Analysis

Cd and Pb concentrations were compared using one-way analysis of variance (ANOVA). The significance of differences between mean values was calculated using Duncan’s test; the differences were deemed significant when *P* < 0.05. The mean values were calculated taking into account three replications for each determination in every sample.

The estimated weekly intakes (EWI) of Cd and Pb were given by the equation:

$$ EWI=\frac{MWC\times metal\kern0.5em  level}{100} $$

where MWC = mean weekly consumption of fishery products.

Two EWI values were calculated: EWI for the actual intake of fishery products in Poland, that is, ca. 112 g per week [13] and EWI(r) for the recommended intake of fishery products, that is, 300 g per week [14].

Tolerable weekly intake % (TWI) and benchmark dose lower confidence limit % (BMDL) were calculated according to the formulas:

$$ \%\kern0.5em TWI=\frac{EWI_{Cd}\times 100}{TWI} $$

$$ \%\kern0.5em  BMDL=\frac{EWI_{Pb}\times 100}{BMDL} $$

The value adopted for TWI was 2.5 μg Cd kg^−1^ of body weight per week [4], while for BMDL two values suggested by the European Food Safety Authority (EFSA) were calculated per 1 week: BMDL_01_–10.5 μg Pb kg^−1^ of body weight per week and BMDL_10−_4.4 μg Pb kg^−1^ of body weight per week [5]. The mean body weight was assumed as 70 kg.

## Results

### The Content of Cd and Pb in Fishery Products

The content of Cd per 1 kg of the analysed product can be represented as follows: salads > smoked eel > smoked salmon and mackerel > smoked trout and spreads > marinated herring > smoked sprat. The highest content of Cd was recorded in salads (on average 10.71 μg kg^−1^; range 6.527–14.70 μg; *P* < 0.004; Table 3). Smoked fish contained on average 6.386 μg Cd per kg (Table 4), with eel containing on average more than 9 μg, and mackerel and salmon nearly 8 μg (Table 3). Fish spreads contained on average 5.2 μg Cd per kg (range < LOQ—10 μg), and marinated fish—3.4 μg Cd per kg (range 0.99–13.98 μg). The significantly lowest content of Cd was observed in smoked sprat (1.263 μg kg^−1^; range 0.755–2.278 μg). The content of Pb per 1 kg of the analysed product can be represented as follows: smoked salmon and salads > smoked mackerel and spreads > smoked eel > smoked sprat and smoked trout. Most Pb (*P* < 0.001) was recorded in marinated herrings (on average 58.81 μg kg^−1^; range 19.81–79.6 μg; Table 3), in fish salads (on average 56.8 μg kg^−1^; range 32.6–78.9 μg), and in smoked salmon (on average 57.8 μg kg^−1^; range 10.97–155.9 μg).

**Table 3:** Results of fishery products analysis

	Smoked					Fish-based dishes			
	Mackerel	Salmon	Sprat	Eel	Trout	Salads	Spreads	Marinated	NOVA *P*
Cd (μg kg^−1^)									
Mean	7.921^c^	7.889^c^	1.263^f^	9.164^b^	5.694^d^	10.71^a^	5.193^d^	3.400^e^	0.004
Maximum	13.44^c^	19.60^a^	2.278^f^	14.40^b^	8.834^e^	14.70^b^	10.02^d^	13.98^b,c^	0.001
Minimum	6.530^a^	<LOQ^e^	0.755^d^	4.445^b^	0.981^c^	6.527^a^	<LOQ^e^	0.993^c^	0.002
Median	0.750	7.170	1.073	8.364	5.756	10.25	4.644	2.078	
SD	1.352	5.880	0.565	3.111	2.311	2.378	3.267	4.399	
Variance analysis	0.184	3.045	0.035	0.974	0.533	0.572	1.071	1.494	
Percentile									
75%	5.063	9.684	2.154	13.41	7.910	13.49	8.842	3.522	
25%	7.278	5.667	0.778	6.878	3.284	8.524	2.321	1.666	
Pb (μg kg^−1^)									
Mean	49.64^b^	57.81^a^	1.964^d^	37.26^c^	1.664^d^	56.82^a^	49.99^b^	58.81^a^	0.001
Maximum	99.64^b^	155.9^a^	3.112^f^	54.59^d^	4.582^e^	78.90^c^	99.64^b^	79.60^c^	0.001
Minimum	10.85^d^	10.97^d^	0.934^f^	8.710^e^	0.491^g^	32.58^a^	15.85^c^	19.81^b^	0.003
Median	41.74	50.78	1.902	41.56	1.292	59.71	40.42	59.50	
SD	1.080	5.243	0.864	15.80	1.322	16.61	24.42	19.72	
Variance analysis	96.58	20.47	0.741	24.98	0.167	27.58	59.65	34.64	
Percentile									
75%	59.64	35.12	2.991	50.68	2.372	77.17	75.29	70.30	
25%	10.97	6.531	1.024	10.79	0.554	33.27	31.91	23.34	

LOQ Cd = 0.004 mg kg^−1^, LOQ Pb = 0.03 mg kg^−1^

*SD* standard deviation, *SEM* standard error of the means, *LOQ* limit of quantitation

^a,b,...g^- mean values in rows with different letters differ significantly at *P* < 0.05

**Table 4:** Safety of fishery products for consumption

	Cd	Pb
Mean content of Cd and Pb (μg kg^−1^)		
Smoked^c^	6.386	29.67
Salads^c^	10.71	56.82
Spreads^c^	5.193	49.99
Marinated^c^	3.400	58.81
Canned^d^	6.895	29.16
Fried and baked^e^	19.01	26.54
Mean level	8.599	41.83
Real fish consumption 112 g [13]		
EWI (μg)^a^	0.963	4.685
% TWI^f,g^	0.550	
% BMDL_01_^f,h^		0.637
% BMDL_10_^f,i^		1.521
Recommended fish consumption 300 g [14]		
EWI(r) (μg)^b^	2.580	12.55
% TWI^h,i^	1.474	
% BMDL_01_^f,h^		1.707
% BMDL_10_^f,i^		4.074

^a^EWI—estimated weekly intake calculated on the basis of the real mean weekly consumption of fishery products and mean level of Cd and Pb

^b^EWI(r)—estimated weekly intake calculated on the basis of the recommended weekly consumption of fishery products and mean level of Cd and Pb

^c^This study

^d^Based on [17]

^e^Based on [16]

^f^Mean body weight was assumed as 70 kg

^g^TWI—2.5 μg Cd per kg of body weight per week [4]

^h^BMDL_01_—10.5 μg Pb per kg of body weight per week [5]

^i^BMDL_10_—4.4 μg Pb per kg of body weight per week [5]

### Cd and Pb Intake with Fishery Products

Assuming that the level of intake of the fishery products by adults is 112 g per week [12], an individual weighing 70 kg consumes 0.96 μg Cd with fish, which does not exceed 0.6% TWI, and 4.7 μg Pb, which does not exceed 1.6% BMDL (BMDL_01_ = 0.64%; BMDL_10_ = 1.52%) (Table 4). Taking into account that the recommended level of intake of fish and fishery products in Poland, that is 300 g per week, an adult would consume 2.6 μg Cd per week (1.47% TWI) and 12.55 μg Pb per week (1.71% BMDL_01_ and 4.07% BMDL_10_) (Table 4).

## Discussion

### The Content of Cd and Pb in Fishery Products

None of the analysed products was found to exceed the admissible standard limit: 0.05 mg Cd per kg for muscle meat of fish, 0.1 mg for eel and mackerel, and 0.3 mg Pb per kg for muscle meat of fish [18, 19]. Comparison of the content of Cd in fishery products measured in the present study shows that the maximum content measured by other authors, based on research carried out in various countries (Table 5), was higher than that determined by the present authors; however, some authors obtained lower results [25]. The results of the present authors obtained for Pb were generally higher than those presented by other authors (Table 5). Sireli et al. [21] demonstrated that the content of Pb in smoked fish ranged from 0.001 to 0.791 mg/kg; these results were higher than those obtained by the present authors, while other authors [20, 22, 23] recorded lower values. Predators accumulate much more toxic metals in their tissues since the metals are capable of accumulating in the organism and are thus transmitted to a higher level of the food chain. Therefore, the likelihood that fish will be contaminated with heavy metals increases with their age [9].

**Table 5:** Comparison of the content of Cd and Pb in fishery products measured in the present study and reported by other authors

		Range, mg/kg fresh weight			
Characteristic	Number	Cd	Pb	Country	References
Smoked fish					
Mackerel, salmon, sprat, eel, trout	139	<LOQ—0.020	<0.001–0.156	Poland	This study
Mackerel, sprat, herring, salmon, trout	60	0.008–0.043	0.002–0.016	Poland	[20]
Mackerel, salmon, trout	73	0.03–0.036	0.01–0.791	Turkey	[21]
Herring	25	0.013–0.043	0.014–0.063	Egypt	[22]
Mackerel, catfish	–		0.02–0.07	Nigeria	[23]
Mackerel, herring, salmon, haddock	4	0.0002–0.0027	No data	France	[24]
Fish-based dishes					
Salads, spreads, marinated herring	117	<LOQ—0.020	0.008–0.100	Poland	This study
Marinated herring, marinated mackerel, salted herring	60	0.010–0.019	0.010–0.019	Poland	[20]
Fish soup, paella, surimi, tarama	5	0.0002–0.015	No data	France	[24]

In the presented studies, the average content of Cd in smoked fish was significantly higher than in marinated fish and spreads, while the level of Pb was significantly lower than in marinated, spreads and salads. Essuman [26] noted that smoked fish contained much more lead than fresh fish did. Igwegbe et al. [27] revealed that fish smoking resulted even in a twofold increase in the content of toxic metals. The authors presented the relationship between these metals as Pb > Hg > Cd > As. Smoking the meat of different species of animals, including fish, is a traditional method of preserving food (smoke has bactericidal and bacteriostatic properties), and it has a positive effect on the organoleptic quality of meat [28]. Regrettably, smoking also adversely alters the chemical composition of meat, e.g. polycyclic aromatic hydrocarbons are formed [29] and the content of toxic metals increases [27]. The resulting smoke is the source of Cd, Pb, As and Hg which partly diffuse into the meat, but most of which is deposited on the product, which is confirmed by the results of tests carried out by Igwegbe et al. [27]. These authors observed that fish after washing contained about 50% less toxic metals than fish that had not been washed. The composition of the smoke is determined by many factors, such as the smoking technology and parameters of the smoking chamber, composition of smoke formulas and type and texture of wood used to produce the smoke [28]. In addition, smoking causes the drying of meat, which in turn increases the concentration of metals in a unit of weight.

Marinated herring and salads as well as fish spreads, according to presented studies, contained significantly more Pb than smoked fish. Ready-made spreads and salads can not only contain fish muscles but also fish livers. Toxic metals are unevenly distributed. Most of them are accumulated in the liver and kidneys [30–32]. The meat of herrings contains more toxic metals than other sea fish [20, 33]. Polak-Juszczak [34] demonstrated that the concentration of Cd and Pb in the waters of the Baltic Sea has decreased over years, which is directly connected with the decreasing concentration of these metals in the meat of the Baltic fish: herring, sprat and cod.

### Safety of Fishery Products

According to EFSA the average weekly exposure of Europeans to Cd is 2.04 μg per kg body weight [4], and to Pb 4.76 μg per kg body weight (0.68 μg kg^−1^ body weight per day) [5]. The level of exposure is higher in children. The weekly exposure to those metals admissible by EFSA is 2.5 μg Cd kg^−1^ body weight. For Pb, this value is 10.5 and 4.4 μg Pb kg^−1^ body weight, taking into account the nephrotoxicity and cardiovascular effect of Pb [4, 5].

The share of fish and fishery products in the supply of Cd and Pb in the diet is connected both with the intake of fish in respective countries and with their content of toxic metals. Studies carried out in Spain revealed that fish on average supply about 1.1 μg Cd and 2 μg Pd per day (7.7 μg Cd and 14 μg Pd per week) to adult men, which were safe values [35]. On the other hand, the Greek population consumes 0.4–0.6 μg Cd day (2.8–4.2 μg Cd per week) with fish and seafood [36]. Studies in Italy showed that the share of fishery products and seafood in supplying toxic metals is, according to different sources, 14–20% TWI Cd and 1.5–14% PTWI Pb [37, 38]. In turn, Cirillo et al. [39] decided that Italians consume on average 0.9% TWI Cd (range 0.1–8.0%) and 2% PTWI Pb (range 0.5–16.5%) with fish. All the above-quoted Italian studies showed that fishery products were safe for human consumption in terms of the content of Cd and Pb. Also, studies carried out in France revealed that eating fish was safe as they supplied adults with 1.94–2.69 μg Cd kg^−1^ body weight per week, depending on the age and the sex [24].

In Poland, the intake of fish is not high. According to surveys carried out by the Public Opinion Research Centre, only 1% of respondents declared they ate fish every day, 29%—a few times a week and nearly half of them (48%)—a few times a month [40]. The presented own studies demonstrated that both the actual consumption (on average 112 g per week) [13] and recommended intake of fish and fishery products (300 g per week) [14] are not hazardous to consumers. Adult Poles weighing 70 kg consume only 0.55% TWI Cd and Pb in the amount of 0.6% BMDL_01_ and 1.5% BMDL_10_ with the analysed products. Previous studies carried out in our team revealed that more than 94% of adult men regularly consumed canned fish, of which 33% ate it at least once a week [17]. Assuming that adult Poles consume a maximum of one tin of fish a week, they consume 0.8% TWI Cd and 1.23% BMDL_01_ and 3.5% BMDL_10_ Pb [17]. In Poland, consumption of fresh and frozen fishery products is about 77 g per person per week [16]. Assuming that an adult consumes 77 g of fried or baked products per week, nearly 19 μg Cd (0.64–0.84% TWI) and 26.5 μg Pb (max. 0.27 BMDL_01_ and max. 0.93% BMDL_10_) will be taken with these products [16]. Taking into account both the share of products presented in own studies and of canned fish, and of fried and baked fishery products, adult Poles consume less than 0.6% TWI Cd and less than 0.7% BMDL_01_ Pb and less than 1.6% BMDL_10_ Pb with these products. These values should be considered safe. According to Usydus et al. [22], with regard to the content of Cd, it would be dangerous for an adult man weighing 70 kg to consume from 11.0 to 63.0 kg of smoked fish a week, depending on the species of fish. At the same time, it should be noted that even for people who are physically active and sportspeople for whom eating fish is recommended a few times a week with regard to the fact that it contains omega-3 acids [41], the risk of excessive intake of Cd and Pb with these products is very unlikely. The most important source of Cd and Pb in the human diet throughout the world is plant products, in particular grains since they are the basic foods throughout the whole world that are consumed most copiously [4, 5].

To sum up, fish and fishery products available in Poland are safe for consumers. Even if fish is consumed at recommended levels exceeding the actual consumption by more than 50%, no hazard exists. The most contaminated products are fish salads (Cd, Pb) and spreads (Pb).
